# A Review of the Dawn of Benchtop EPR Spectrometers—Innovation That Shaped the Future of This Technology

**DOI:** 10.3390/molecules27185996

**Published:** 2022-09-14

**Authors:** Vasily V. Ptushenko, Vladimir N. Linev

**Affiliations:** 1 A.N. Belozersky Institute of Physico-Chemical Biology, Lomonosov Moscow State University, 119992 Moscow, Russia; 2 N.M. Emanuel Institute of Biochemical Physics, Russian Academy of Sciences, 119334 Moscow, Russia; 3 LINEV Group, LINEV Systems UK Limited, London SW1Y 5JG, UK

**Keywords:** EPR instrumentation, geologic exploration, chemical industry applications, EPR dosimetry, Belarusian State University

## Abstract

By the early 1980s, unique devices appeared in the USSR: a series of benchtop specialized EPR spectrometers. This equipment was quickly accepted not only in science but also in medicine and in many technical and economic areas including chemical industries and geologic exploration. The appearance of these devices was perceived as a salvation for the Soviet magnetic resonance (MR) scientific instrumentation by those who worked in the field of EPR spectroscopy in the USSR. (However, the program of MR scientific instrumentation ceased to exist along with the USSR a few years later). The Belarusian State University in Minsk was the center of these developments. At that moment and for many years afterwards, these devices were unique with no analogues in the worldwide EPR industry. They remained the only mass-produced MR spectrometers on the territory of the former USSR after its collapse. For the first time, based on archival materials, patents, and our personal memoirs, we describe the development of these EPR spectrometers and discuss the most original technical solutions and the scientific tasks solved with this equipment We also remember the participants of the work, showing the historical context of these events.

## 1. Introduction

In this paper, we describe the history of the creation of a series of scientific instruments, which is work that involved many people and research teams and played a significant role in scientific research in the field of EPR spectroscopy in the late 1970s–1980s and contributed to the USSR economy. However, the work involved in the creation of innovative scientific instruments, whilst essential for progress in science, often remains unnoticed by the scientific community, which is aware of only the final result. Still, a “master’s tool” has been a subject of interest in all ages. We believe that this applies to the work and innovation of the EPR spectroscopy instruments. 

This particular story has never been described in detail before; only a few early episodes have been reflected on to some extent in memories and discussions. In addition, despite the vast amount of work involved and the fact that a significant number of patents were obtained, virtually no articles were published. Therefore, to fill this gap, in this article, we describe the work involved based mainly on archival materials, patents, and personal memoirs of one of the authors. As this is the first description ever published, we dare to go into some historical details, focusing not only on the scientific tasks being solved but also on the technical details of the devices being developed as well as on the people participating in this work. We believe that the published memoirs of J. Hyde [1], who did not consider it shameful to include aspects of live communication between colleagues while telling about the technical history of “Varian”, serve as a good example of how this mix of factual and personal aspects works well.

The history of the development of EPR spectrometers in the USSR described here is not an isolated one “without kith or kin”. It is inscribed in the context of many years of research, development and instrumentation in the field, which began with the discovery of the EPR by Eugene K. Zavoisky in 1944 [2,3]. Some previous stages of the development of EPR spectrometers in the USSR were described in our earlier publications [4,5,6,7]. In this paper, we describe the last stage in the USSR experimental design program in the field of EPR, which later found continuation in the post-Soviet history of EPR instrumentation. The development appeared on the background of significant stagnation in the development and serial production of research-grade EPR spectrometers in the country which had become obvious by the mid-1970s. 

The leaders in the field were Varian (USA) and Bruker (Germany), who developed and produced a set of unique EPR spectrometers which became state of the art standard for EPR spectroscopy. It was impossible to achieve the same level of instrumentation in the USSR with the outdated electronic components then available. Therefore, the salvation of Soviet EPR spectroscopy capability in these conditions required an innovative and creative approach rather than conventional design and technical solutions. 

It is worth noting that although the article addresses the history of applied research, it remains relevant to the issues of modern economic theory of high-tech innovations, especially Christensen’s Theory of Disruptive Innovation. The Department of Nuclear Physics of the Belarusian State University, where the development of benchtop EPR spectrometers started, was decades ahead of its time in terms of its scientific and institutional concept to innovation and development processes. In fact, it was an innovative incubator, i.e., a generator of high-tech ideas, which then resulted in establishing scientific laboratories, research institutes, and design centers. 

The benefits of this scientific and economic model are confirmed, in our opinion, by the emergence of a group of high-tech companies including the LINEV Group, the producer of specialized benchtop EPR spectrometers, which considers itself the heir to the scientific and innovative traditions of the Department of Nuclear Physics. 

## 2. Historical Background for the EPR Instrumentation in Minsk 

**Figure 1:**
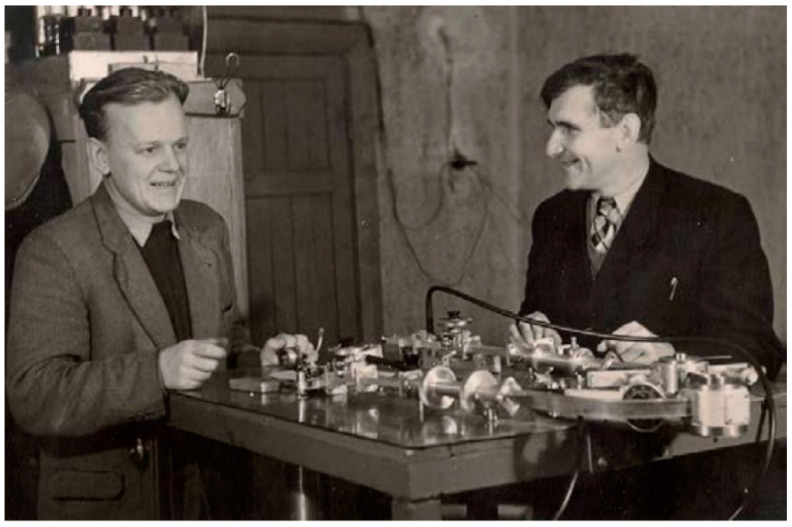
Mikhail A. Eliashevich (**right**) with his disciple Stanislav S. Shushkevich (**left**) near the microwave assembly of their handmade EPR spectrometer. Minsk, 1957. Source: personal archive of V.N. Linev.

In the 1950s, the main centers of EPR spectroscopy in the USSR were the Kazan Physical-Technical Institute (PTI; now E.K. Zavoisky PTI), the Moscow P.N. Lebedev Physical Institute (LPI), and the Moscow Institute of Chemical Physics (ICP; now N.N. Semenov ICP) of the Soviet Academy of Sciences (AS). The roots of the EPR instrumentation in Belarus were connected with these scientific centers. In 1956, the famous physicist Mikhail A. Eliashevich (1908–1996) moved to Minsk from the ICP in Moscow. In the ICP, chemical and biological EPR spectroscopy had been being developed since 1954, and Eliashevich was in the middle of events and actively participated in them [7]. In 1956, Stanislav Stanislavovich Shushkevich became his postgraduate student at the Institute of Physics and Mathematics of the Academy of Sciences of the Belarusian Soviet Socialist Republic (BSSR) (Figure 1). (Much later, in the 1990s, he became known as the head of Belarus who signed the Belovezh Accords with M.S. Gorbachev declaring that the USSR had effectively ceased to exist). 

Eliashevich set his new laboratory team the task of experimentally mastering the detection of electron and nuclear magnetic resonance (EPR and NMR) and nuclear quadrupole resonance (NQR) spectra. To solve this problem, in early 1957, postgraduates S.S. Shushkevich and A.K. Potapovich went on an internship sat the LPI (specifically at the laboratory of A.M. Prokhorov, the future Nobel laureate) and ICP where the problem had been already had solved ([8], pp. 56–58). 

In 1952–1953, Prokhorov, on behalf of the LPI director D.V. Skobeltsyn, switched to work that eventually led to the appearance of EPR masers [9]. For this purpose, A.A. Manenkov was transferred to LPI from Kazan PTI. In Kazan, A.A. Manenkov was a graduate student of B.M. Kozyrev, a colleague of E.K. Zavoisky [10], the discoverer of EPR. Thus, the EPR arrived in LPI from the “original source”. At the same time, in the ICP, V.V. Voevodsky and L.A. Blumenfeld with their colleagues were developing EPR spectrometers suitable for chemical and biological works. The communication of Shushkevich and Potapovich with these scientific groups and the study of their experience made it possible to create in Eliashevich’s laboratory in Minsk in the next three years a superheterodyne ESR spectrometer with two five-ton electromagnets made for this at the Leningrad “Electrosila” factory ([8], pp. 58–59).

## 3. Development of Benchtop EPR Spectrometers: The First Steps

Having experienced the influence of key figures in EPR spectroscopy in late 1950s, the development of EPR instrumentation in Belarus bloomed and led to the concept (as well as the “incarnation”) of small-sized EPR spectrometers a decade and a half later, when Shushkevich was already working at the Belarusian State University (BSU) as the head of the Department of Nuclear Physics and Peaceful Uses of Atomic Energy. His department, including the teaching staff and research laboratories, was the largest at the Physics Faculty of BSU (about 120 people) and, during its life, gave birth to several institutes and Special Design Departments (SDD). It specialized in applied instrumentation in the field of nuclear physics. Despite the obvious significance of this work today, its beginning was prompted by a rather accidental circumstance, namely, the attempts of the neighboring Department of Semiconductor Physics to develop a small-sized device for educational purposes. These attempts were based on the dead-end (as it turned out later) idea of using the pole tips of an electromagnet as the walls of a resonator. Actually, this approach did not allow obtaining a high Q-factor and hence high enough sensitivity of the resonator. V.F. Stelmakh, the head of the Department of Semiconductor Physics, asked Shushkevich to help with this work. The actual work was started in 1975 by one of the authors of this article, V.N. Linev, who had become a department member shortly before then. This task became the topic of his PhD thesis and later of his doctoral dissertation. (In the USSR, there was a two-stage system of academic degrees.) 

By 1978, manufacturing of the first device was completed. It was housed in an oscilloscope case of ca. 274 × 206 × 440 mm^3^ size. Compare it with RE1306 EPR spectrometer—the main mass-produced EPR spectrometer in the USSR at that time—which consisted of four blocks. One of them, the electromagnet, was 1290 × 1255 × 710 mm^3^ in size; the other three blocks were slightly smaller but comparable in size [11]. Hence, the new equipment drastically surpassed existing models in compactness; however, its capabilities were limited. 

It was obvious that to create a full-fledged scientific device rather than one serving for educational purposes only, significant efforts were still required. However, such a work demanded funding. Furthermore, before starting the development, it was necessary to assess the need for benchtop EPR spectrometers of Soviet science and industry. S.S. Shushkevich had a very wide network of acquaintances in Soviet radio spectroscopy, and he helped Linev to get in contact with some specialists in EPR spectroscopy who became the key customers and investors to drive the whole project. In Moscow, Linev met L.V. Bershov from the Institute of Geology of Ore Deposits, Petrography, Mineralogy and Geochemistry (IGEM) of the Academy of Sciences of the USSR and B.M. Moiseev from the All-Union Scientific-Research Institute of Mineral Resources (VIMS) of the Ministry of Geology of the USSR. 

These institutes were engaged in the exploration of uranium deposits [12,13]. Leonid V. Bershov studied paramagnetic centers in minerals; he showed that they could be used in petrographic research to study the diastrophic blocks, e.g., determining their radiation history and conditions of their formation as a whole (see, e.g., [14]). Boris M. Moiseev studied paramagnetic Al- and E-centers in quartz crystals and determined the rate of their formation due to the radiation of different types resulting from the decay of uranium, thorium, potassium and other radioactive nuclei contained in the rock. This made paramagnetic properties of rocks an indicator for exploration of uranium deposits [15,16]. Thus, a new direction of paleodosimetry, a paleodosimetric method of searching for uranium deposits, was opened in geology. The detection of radioactive uranium ore lying at considerable depths by studying paramagnetic centers in the rocks at the surface layers thus became possible, and EPR allowed the delimiting of uranium deposits. However, the outdated models of EPR spectrometers adjusted for laboratory work, e.g., RE1301 [14] or RA-100 [15], remained the main instruments of these studies. The development of small-sized portable EPR spectrometers suitable for work in expeditions would significantly reduce the cost of and speed the exploration of minerals. Finally, L.V. Bershov and B.M. Moiseev found funding for the development of small-sized EPR spectrometers. 

Several contracts were agreed between BSU and VIMS on the basis of long-term cooperation for at least 10 years in the future. An industrial scientific-research laboratory for magnetic resonance and gamma resonance spectroscopy of geologic materials was established in 1980 to carry out the development of small-sized EPR spectrometers. In 1985, V.N. Linev was appointed head of the laboratory [17]. Formally, since ca. 1977 until 1991, the laboratory developed and manufactured EPR equipment (e.g., [18]), as well as a wide range of other scientific instruments, for the Ministry of Geology of the USSR (Mingeo). Thus, for the first time ever, portable automated Mössbauer spectrometers (used for analysis of cassiterite, the main ore mineral for tin production, in ores) were developed, and the experimental factory of BSU allowed the manufacturing of a series of these devices. 

We have covered these organizational issues to show the motives and method of organizing such a study in the USSR where direct commercial activity was impossible. Actually, the main challenges were related to innovating new technical ideas that were essential to achieve compact spectrometers. To some extent, the developers learned the technique of EPR instrumentation by studying the design of Varian and Bruker spectrometers available in the USSR. Copying these devices was out of the question due to the lack of necessary modern electronic components in the USSR coupled with the realization at an early stage by V.N. Linev that the simple miniaturization of the full-size spectrometers developed earlier was a dead-end. Compact devices had to be developed “from scratch” with essentially new solutions for each component of the instrument. Surprisingly, this project benefited from the fact that its participants were “amateurs” in the field of EPR spectroscopy. They were not “blinded” by conventional technical solutions, and this meant that they were able to innovate new solutions to the technical challenges. 

## 4. Technical Solutions for Compactization of EPR Spectrometers

By far, the most massive element of a full-size EPR spectrometer is the electromagnet. In the most common Soviet X-band EPR spectrometer of that time, RE1306, the electromagnet weighed more than a ton, and for the next model RE1307, it was ca. 3.5 tons. For a portable instrument, it should not exceed 40–50 kg. The mass of the electromagnet grows rapidly with the increase in the size of the pole gap in which a uniform magnetic field must be provided. Hence, reducing the electromagnet mass by almost two orders of magnitude should be the first step toward creating a benchtop device, and this reduction depended on the minimum possible pole gap width. We mentioned above the attempt to use the pole tips of the electromagnet as the walls of the resonator. This challenging idea (called “EPR sensor”) allowed a pole gap as narrow as 8 mm, but this approach turned out to be a dead end. The requirement to achieve a sufficiently high resonator Q-factor was incompatible with a monolithic “magnet-plus-resonator” design and demanded at least a 14 mm pole gap. According to this minimum distance and the requirements for maximum magnetic field inhomogeneity (≤10^−5^), various systems to create and customize a magnetic field were developed.

**Figure 2:**
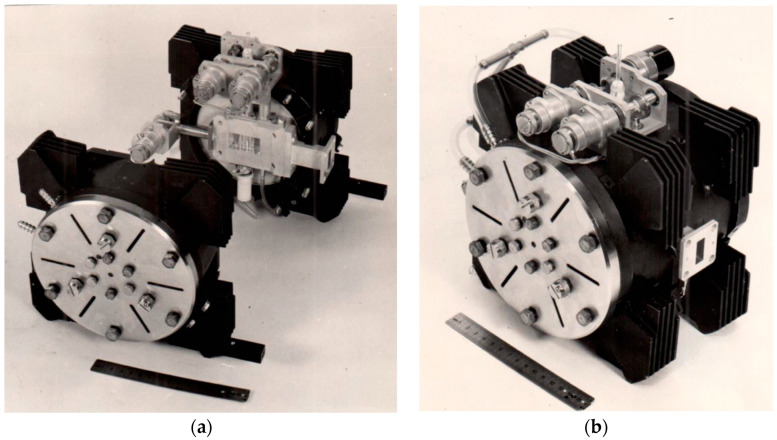
EPR resonator combined with the synchronous detector, dismantled (**a**) and assembled (**b**). Source: Personal archive of V.N. Linev.

**Figure 3:**
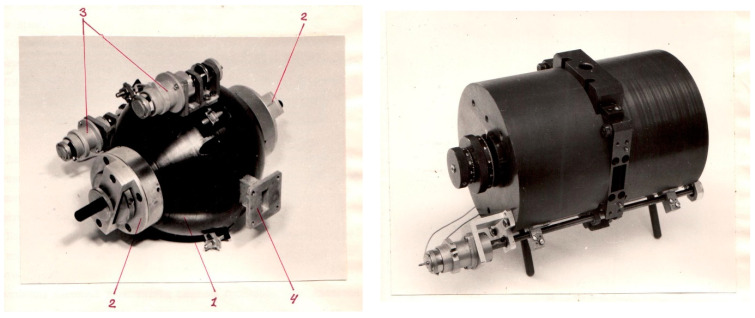
Two models of permanent magnet-based magnet blocks for benchtop EPR spectrometers. A piece of waveguide of X-band EPR spectrometer in the left photo indicates the miniature size of the magnet. The diameter of cylindrical magnet block in the right photo is 14 cm. *1*—magnet yoke, *2*—magnetic field adjustment mechanism, *3*—frequency tuning and coupling nodes of the measuring resonator, *4*—lead-in waveguide. Source: personal archive of V.N. Linev.

Further technical findings involved using flat rectangular plates for magnetic field modulation and a modular resonator [19]; the former provided reducing the size of the resonator without reducing its Q-factor, while the latter allowed adapting the resonator to different tasks. This extraordinary design required taking into account the distribution of electric and magnetic fields along the assumed cutting lines of the resonator. The resonator was combined with a synchronous detector of original design (Figure 2). The replacement of the electromagnets conventionally used for EPR spectroscopy by permanent magnets-based systems was probably the most paradoxical technical solution (Figure 3). This solution allowed a reduction in the mass of the magnet by several orders (down to 8 kg!) and the energy consumption by an order of magnitude. 

**Figure 4:**
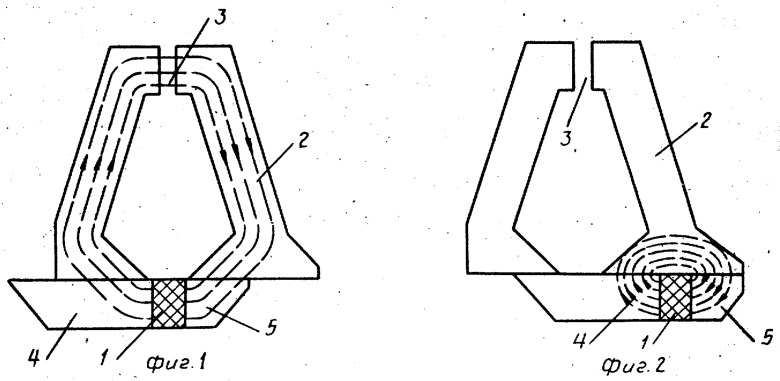
The scheme of mechanical regulation of magnetic field in the pole gap employing moving the additional permanent magnet relative to magnetic circuit [21]. *1*—permanent magnet, *2*—magnetic circuit, *3*—pole gap, *4*,*5*—movable part of magnetic circuit. This scheme provided a change in the magnetic field in the pole gap in the range 0.01–0.7 T.

To regulate and sweep magnetic field ***H*** in the pole gap, various methods of mechanical and electro-mechanical ***H*** regulation were invented, employing moving the additional permanent magnet relative to the magnetic circuit, introducing additional adjustable gaps into the magnetic circuit, etc. [20,21,22,23] (Figure 4). This branch of experimental design was supervised by V.V. Lisovsky, a member of the BSU Department of Nuclear Physics. The electronic computing facilities available at the BSU at that time were used for mathematical simulation that preceded the experimental work. Along with permanent magnets-based EPR spectrometers, the electromagnet-based lineup was developed. The design of a compact electromagnet providing highly homogeneous magnetic field comprised modeling of the magnetic circuit and the pole tips configuration as well as the search for a material with optimal characteristics. 

The other massive component of the EPR spectrometer, a power source, was reduced significantly in size due to a reduction in the energy levels required. In a conventional EPR spectrometer, the electromagnet and microwave source (klystron) are among the main energy consumers. As the energy demand of the magnet was reduced, the klystron energy requirements became the limiting factor. To solve the issue, the time-tested klystron (which bore all the disadvantages of electro-vacuum devices) was replaced by a Gunn diode, which was a solid-state electronic element invented a decade earlier. The original Gunn diode-based X-band microwave generator, highly stable and tunable in frequency, was probably one of the key elements invented already at the start of the project and subsequently being improved over the following years. The second approach was to use a p-i-n diodes-based microwave attenuator. These inventions allowed reducing the total mass and energy demand of the spectrometer as well as improving the microwave durability. 

To simplify the use of the device, automatic tuning systems were developed for all the units. One of these was an approach later called the Method of Adaptive Registration of EPR spectra. This involved sweeping the magnetic field with a varying speed depending on the spectrum slope [24]. This allowed skipping “empty” regions (free of EPR signals) during ***H*** sweeping without manual tuning of recording parameters and hence significantly increased the efficiency of recording multicomponent spectra (especially with long-term signal accumulation). The techniques for stabilization of the magnetic field and resonance conditions during long-term continuous measurements were developed as well as for on-the-fly signal accumulation and processing, which was necessary for EPR recording in the field [20,25]. All these solutions, in different combinations depending on the specific requirements and tasks, were embodied in a whole series of small-sized EPR spectrometers developed in the BSU Industrial scientific-research laboratory for magnetic resonance and gamma resonance spectroscopy of geologic materials in subsequent years. 

Below, we describe the scope of application of small-sized EPR spectrometers and how it expanded (Section 5). However, each class of applications required the development of special technical solutions. As an example, a special resonator for magnetic semiconductor materials with magnetic bubble domains, a subject of great interest for the electronics industry, was developed (in fact, it was a ferromagnetic resonance (FMR) spectrometer). The size of the samples (usually large plates or films) did not allow them to be placed in the resonator. An alternative design of measurement techniques was developed instead: the sample was placed outside the resonator, and a coupling element between the sample and the resonator was inserted inside the latter. The sample holders provided precise movement of the sample relative to the coupling hole, which allowed obtaining a profile of magnetic defects in the sample. Thus, an FMR scanner for magnetic films was developed. 

For biological samples, a special model of the resonator was also developed. It included a thick-walled ceramic or quartz tube of large diameter, which allowed partially shielding of the electric component of the field in the resonator. Resonators with one-, two- and three-axis goniometers were constructed for studying paramagnetic centers in crystals in IGEM. The built-in goniometers allowed precise positioning of the crystals inside the resonator. In addition, there was a method for measuring the anisotropy parameters of paramagnetic samples employing an alternating modulating magnetic field perpendicular to the polarizing magnetic field [26]. 

**Figure 5:**
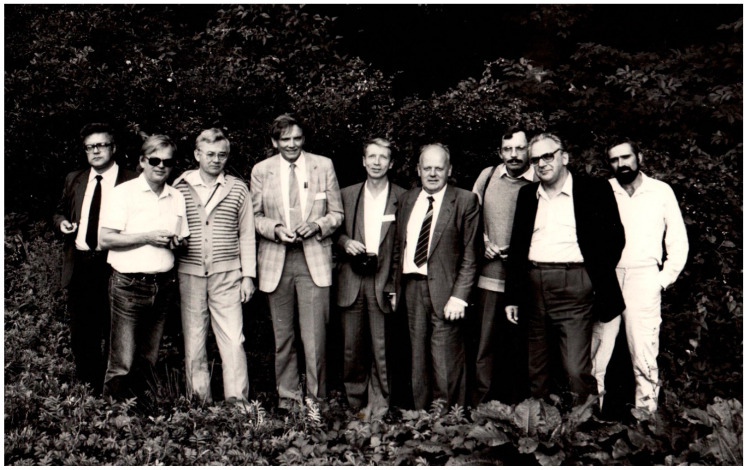
Among the participants of the XXIV Congress AMPERE on magnetic resonance and related phenomena in Poznan, 1988: J. Hyde (4th from the left), Yu.V. Yablokov (in the center), S.S. Shushkevich (4th from the right), V.N. Linev (3th from the right). Source: personal archive of V.N. Linev.

Eventually, a complex of original technical solutions for adjusting each of the nodes of the EPR spectrometer design for specific applications was developed [27,28]. The experience gained actually turned the BSU Industrial laboratory into one of the national-scale centers for the development and production of EPR spectroscopy equipment (Figure 5). 

This detailed description of specific technical solutions is interesting in understanding the development of technology as a whole. Here, we can compare some different approaches in the drift of technical ideas. The need for benchtop EPR spectrometers was quite obvious. Two other groups of developers from the V.I. Ulyanov (Lenin) Leningrad Electrotechnical Institute (LETI) and from the Leningrad Association of Electronic Instrumentation “Svetlana” were solving similar challenges, i.e., the development of small-sized EPR spectrometers for oil production and refining. However, they started from “classic” EPR spectrometer design and moved toward its miniaturization. Probably, this approach based on the “scaling” of full-sized devices had more severe limitations. Thus, the minimum weight of the EPR spectrometer with this approach was about 75 kg (cf. 28 kg achieved in BSU) ([29], pp. 11, 20). 

## 5. New Applications of Benchtop EPR Spectrometers

As the development progressed, the contacts of developers with potential users expanded, and positive feedback led to new applications of small-sized EPR spectrometers. One of the most fruitful came from the contact of V.N. Linev with Peter M. Solozhenkin, who was one of the leaders of the Academy of Sciences of the Tajik SSR. Tajik industry needed an instrument to control ore cleaning at metallurgical plants, and in 1978–1979, BSU produced a benchtop EPR spectrometer for this purpose. This work served as a catalyst for expanding the scope of benchtop EPR spectrometers. The developers started collaboration with Yuri A. Zolotov and his group from the V.I. Vernadsky Institute of Geochemistry and Analytical Chemistry (GEOCHI) of the Soviet Academy of Sciences. The interests of GEOCHI included the chemistry and geochemistry of uranium [30]. Although Yu. A. Zolotov had already collaborated with another EPR spectrometers manufacturer, the Leningrad Electrotechnical Institute [31], the new devices from BSU proved to be in demand. A while later, they proved to be in demand in Zelenograd, the Soviet “Silicon Valley”, where FMR could be used to control the quality of materials with magnetic bubble domains (we described the development of an FMR scanner in the previous section). In Zelenograd, Anatoly Yu. Kozhukhar coordinated this collaboration [32,33]. 

An important direction in the EPR equipment development appeared due to BSU collaboration with Oleg P. Revokatov, Yuri M. Petrusevich, and their coworkers from Moscow State University. O.P. Revokatov suggested an idea to detect the cancer risk using spin probes in a blood test. Previously, Revokatov tried to employ NMR data on proton spin-lattice relaxation in human blood serum as a cancer diagnostic method [34], and he participated in the development of NMR equipment for the diagnostic testing of biological liquids. The new idea was based on employing the rotational mobility of spin probes (which is reflected in the EPR spectra) for assessing the formation of permolecular structures in blood. 

All this work assumed clinical usage and hence required, in addition to compactness, easy-to-operate (i.e., automated) devices. Together with Shushkevich, Revokatov managed to interest the Belarusian Research Institute of Oncology and Medical Radiology (later awarded the name of N.N. Alexandrov and transformed to National Cancer Centre of Belarus”) and, personally, Alexander A. Mashevsky and Violetta V. Prokhorova, in this idea. After the Chernobyl disaster in 1986, the collaboration with physicians transformed into studies on EPR dosimetry. Although this collaboration did not lead to any joint publications, the method for diagnosing malignant neoplasms of lungs using spin labels and benchtop EPR spectrometer RM-10-40-RD was developed in the N.N. Alexandrov National Cancer Centre of Belarus [35]. It also stimulated to some extent the further development of EPR dosimetry in Belarus [36]. Motoji Ikeya, a Japanese physicist known for his contribution to EPR dating [37] and dosimetry [38,39], visited BSU Industrial Laboratory (mainly transformed to ADANI company by then) in Minsk in the early 1990s to review new EPR equipment and met with one of the authors of this article (V.N. Linev). 

## 6. Conclusions

**Figure 6:**
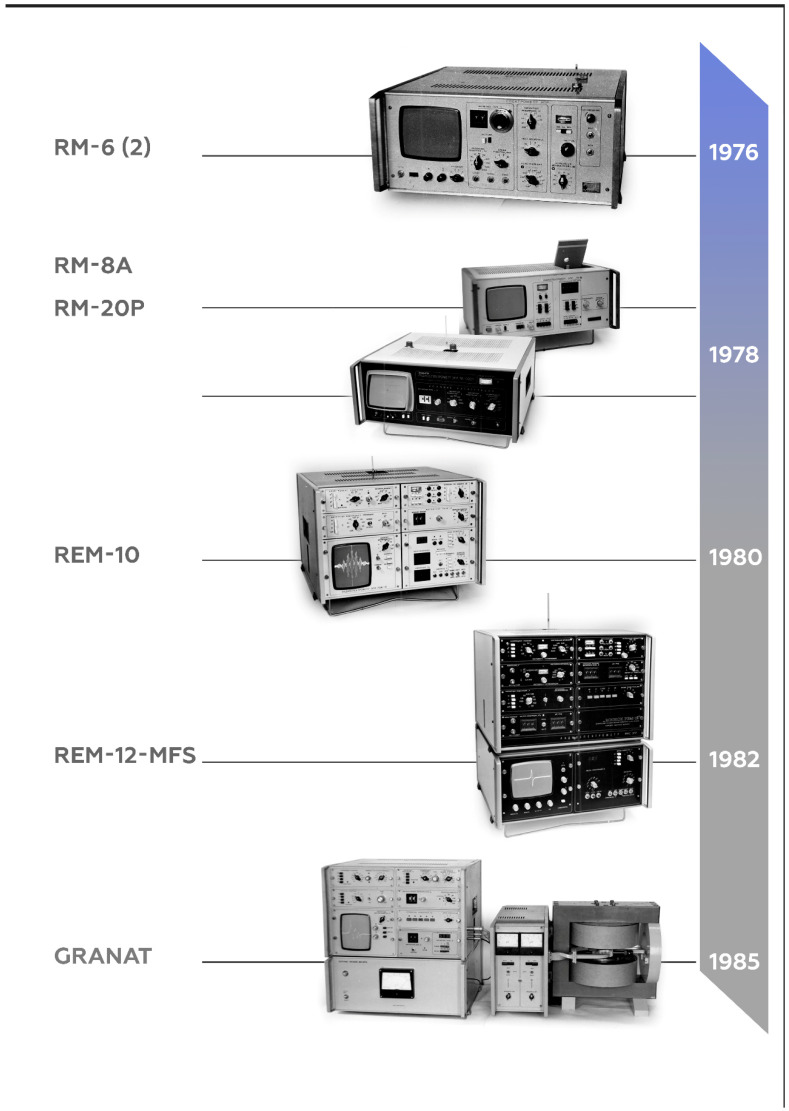
Family of benchtop EPR spectrometers developed and serially produced (in small series) by 1985.

The diversity of applications resulted in wide range of specialized models of EPR spectrometers. At least nine models were developed and serially produced by the mid-1980s including the RM-6, RM-20P, REM-10, REM-12MFS, REM-10-30, REM-10-31, REM-10-32, REM-10-33, and REM-10-35 models (Figure 6). Three of them, RM-6, REM-10 and REM-10-31, were used in research in geology for paleodosimetry and the detection of deep-lying ore bodies. Their use substantially changed the design of exploration expeditions, allowing completion of the exploration within one season. RM-20P served for technological control of ore enrichment in non-ferrous metallurgy (instead of more time-consuming chemical methods used before). An REM-10-33 EPR spectrometer was developed and passed clinical testing for the early diagnosis of oncological diseases using spin labels. Serial mass production of an REM-10-32 universal EPR spectrometer was launched under the trade name AE-4700 in Lviv (industrial group “Micropribor”). In the late 1980s, AE-4700 was the only commercially produced EPR spectrometer in the USSR. 

In addition to the above, in the chemical industry, benchtop EPR spectrometers were used to build an automated control system for preparing a polyisoprene polymerization catalyst; in the non-ferrous-metals industry, to develop methods of quantifying the micro-content of 27 elements; in geology, to elaborate methods for evaluating and searching for deposits of non-ferrous and rare metals; in the oil industry, to provide reliable and safe methods for monitoring the movement of formation water in oil fields; in microelectronics, to introduce non-destructive methods for measuring and certifying magnetic thin films; and in agriculture, to control infestation of grain by pests ([29], pp. 2–3) amongst other applications. 

In summary, the impact of benchtop EPR spectrometers (developed in BSU, LETI and “Svetlana”) on Soviet economics was assessed as already exceeding some 5 million rubles (i.e., some $7.5 M) per year by the early 1980s ([29], p. 3). Hence, the series of benchtop EPR spectrometers was awarded several prizes, the most major of which was the prize of the Council of Ministers of the USSR in 1985 (all three groups of developers of benchtop EPR spectrometers, from BSU, LETI and “Svetlana”, were awarded). That was one of few prestigious awards in the USSR concerning EPR. 

However, along with the industrial applications listed above, these devices were no less important for radiospectroscopy in the USSR. The tragedy for Soviet radiospectroscopy was that the serial production of EPR spectrometers in the USSR was stopped from 1984. Given the difficulty of buying scientific equipment abroad, this had a detrimental effect on science in the USSR. Erlen I. Fedin, who headed the Commission on radio spectroscopy of the Soviet Academy of Sciences and dedicated his life to improving Soviet NMR/EPR instrumentation [5], hoped that this last Soviet EPR developmental project could save radiospectroscopy in the USSR from disaster and provide science and industry with much needed EPR equipment ([29], pp. 162–163). Although it did not save the Soviet EPR instrumentation, the new conditions in the post-Soviet space helped to revive it to some extent in due to the work of the “ADANI” company. 

In this paper, we have given a brief history of development of the benchtop EPR spectrometers in the USSR. For the Soviet science, this project was a chance to “revitalize” EPR spectroscopy studies which were being suffocating due to the lack of modern instruments. 

However, considering the broader context, this history could cause innovation for the future. In our opinion, benchtop EPR spectrometers production could be a kind of “disruptive innovation” in the sense of Clayton Christensen, i.e., the one that creates a new market albeit initially inferior to the current favorite in terms of operating capabilities [40]. 

**Figure 7:**
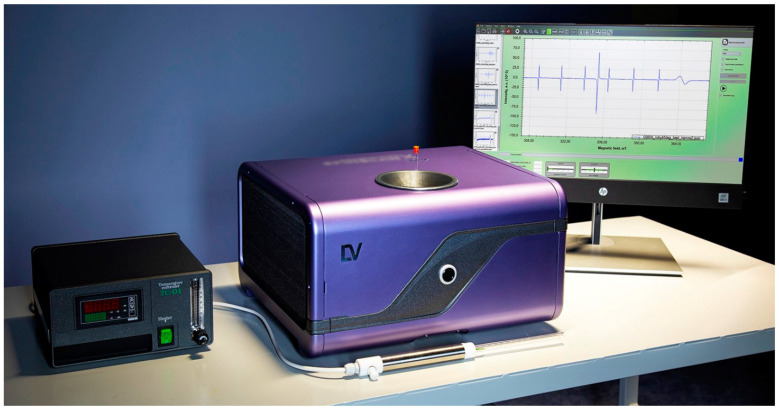
The appearance of a modern benchtop X-band EPR spectrometer (the 2022 model).

This means we can expect that, in the near future, benchtop EPR spectrometers will conquer the market and, in a sense, will displace the research-grade EPR spectrometers as well as smartphones have displaced the professional photo and video cameras. Already now, there are more than 500 modern benchtop EPR spectrometers operating around the world (Figure 7). New technologies in microelectronics make it possible to find even more compact and low-cost technical solutions for all nodes of the EPR spectrometer than those described above. Although they may not in the immediate future be able to compete with research-grade EPR spectrometers in scientific studies, their cheapness and ease of use can significantly expand the scope of their application in the future, and ongoing innovation will enhance their performance step by step. 

## Data Availability

The data analyzed in this study are available from the authors upon reasonable request.
